# Evaluation of biochemical composition of hulled and hull-less genotypes of pumpkin seeds grown in subtropical India

**DOI:** 10.1016/j.heliyon.2023.e12995

**Published:** 2023-01-19

**Authors:** Ananaya Charaya, Neena Chawla, Ajmer Singh Dhatt, Madhu Sharma, Sanjula Sharma, Inderpal Kaur

**Affiliations:** a Department of Biochemistry, Punjab Agricultural University, Ludhiana 141004, India; b Department of Vegetable Science, Punjab Agricultural University, Ludhiana 141004, India; c Directorate of Research, Punjab Agricultural University, Ludhiana 141004, India; d Department of Plant Breeding and Genetics, Punjab Agricultural University, Ludhiana 141004, India

**Keywords:** Pumpkin seeds, Protein, Polyunsaturated fatty acids (PUFA), Polyamines, Acetyl CoA carboxylase, Nutraceuticals

## Abstract

Pumpkin seeds are one of the functional foods with most potential having myriad of uses, and functioning as both edible seeds and oilseeds. Nevertheless, their utilization is restricted to the presence of a thick seed coat (hull) which subjects them to the process of decortication, increasing the farmers’ expense as well as limiting their utilization as oilseeds. Therefore, in the present study, characterization of the biochemical composition of the hulled (Punjab Chappan Kadoo-1 abbreviated as PCK-1) and hull-less (PAU Magaz Kadoo-1 abbreviated as PMK-1) genotype of pumpkin seeds was undertaken to assess the nutritional differences and their efficient application; PMK-1 is a new cultivar of pumpkin released by Punjab Agricultural University in 2018. Based on the characterization, the hulled genotype of pumpkin seeds was observed to possess higher content of total soluble proteins (79.62 mg/100 g), total free amino acids (3.48 g/100 g), moisture (6.74%), fibre content (21.1 g/100 g), antioxidant potential (26.15%), polyamines (19.2 mg/100 g), sterols (387.1 mg/100 g), and specific enzymatic activity whereas the hull-less genotype was observed to possess a higher amount of minerals (4.57 g/100 g), tocopherols (15.76 mg/100 g), and oil content (36%) respectively; most of the biochemical parameters do not differ from each other at a greater fold difference except for total free amino acids and fibre, which are nearly four times and three times higher in hulled seeds in comparison to the naked seeds respectively. The two genotypes of seeds do not compete, rather do complement each other in biochemical and nutritional composition.

## 1 Introduction

The growing globalization, urbanization and increasing income levels have resulted in an upsurge in incessant demand for food or food-based products play a crucial role in their contribution to the food and nutritional security of the people in underdeveloped as well as developing countries, as they are rich in vitamins, minerals, dietary fibre, and phytochemicals. Nevertheless, the current fast-paced life of working individuals or the low nutritional status of the children or aged population in underdeveloped/developing countries does not allow them to consume well-cooked healthy food. Therefore, an alternative supplement with attributes of being inexpensive, nutritious and easily accessible, becomes quintessential to population of each stratum of society.

Pumpkin (*Cucurbita* sp.), of genus *Cucurbita* and family *Cucurbitacae,* is a fruit vegetable-a large, diploid plant with twenty pairs of chromosomes. It is believed to be native to North America and is one of the primary domesticated crops [1,2]. Pumpkins were primarily cultivated for their seeds because the flesh of wild Cucurbita species was inedible owing to its bitterness, thus, seeds were the first to be consumed by human beings [3]. The hulled seeds have a thick hard protective covering known as seed testa or hull [4]. Inside the seed coat, five disparate layers (epidermis, hypodermis, sclerenchyma, parenchyma, and chlorenchyma) undergo lignification to form seed testa [[5], [6], [7]] while in the hull-less species, the outer four layers collapse into a hyaline sheath, resultantly leaving no traces of lignin in the testa and leading to visibility of the high content of protochlorophyll in the innermost layer resulting in olive green colour of the seeds [5,[8], [9], [10], [11]]. The oil extraction entails manual decortication of the thick seed coat of hulled seeds. However, the mutant species, hull-less *Cucurbita pepo* subsp. Pepo cultivated between 1870 and 80, at the Austro-Hungarian Monarchy [12], was efficient in oil extraction, yielding quality vegetable oil, making pumpkin an oil crop [13,14].

Pumpkin seeds, regarded as by-products, discarded as household and agro-industrial wastes, have a plethora of nutritional properties, thereby, they possess the potential to act as nutraceuticals. The presence of biologically active compounds like proteins, oils, sterols, polyamines, and antioxidants, to name a few; have secured pumpkin seeds a position in the traditional medicine system [15]. The seed proteins play a pertinent role in ameliorating health problems in various ways like curbing depression; being rich in: amino acids acting like neurotransmitters, especially l-tryptophan, citrullin, ethylasparagine and γ-amino butyric acid (GABA) [16,17]; anti-thelmintic globular proteins like cucurbitin and carboxypyrrolidine (structural analogue of proline) [18], anti-cancerous by inhibiting the growth of melanoma cells M21 by a protein isolated from *Cucurbita moschata,* known as ribosome inactivating protein (RIP) [19] as well as inhibiting the proliferation of leukemia K-562 cells by a basic protein known as Myeloid Antimicrobial Peptide (MAP) [20]. The seeds are exquisite sources of saturated and unsaturated fatty acids where palmitic acid, stearic acid, oleic acid, and linoleic acid predominate and comprise more than 98% of all fatty acids [14]. The presence of other metabolites like tocopherols, tocotrienols, β-carotene and phenolic compounds contribute to their high antioxidant potential. Another significant metabolite, β-sitosterol, a phytosterol is well known to inhibit and slow down the growth of the cancer cell lines in the colon, lungs, stomach, ovaries, and breasts. β-sitosterol is also well known to be used in the cure for prostate hyperplasia [13,21]. The seeds are not only nutritionally relevant rather, but they also behave as nutraceuticals validating Hippocrates statement “***Let food be thy medicine***”, a statement made around 2000 years ago. The term “nutraceutical” is defined as “a food (or part of a food) that provides medical or health benefits, including the prevention and/or treatment of a disease."

According to market estimates, the economic value of the seeds is expected to surge in the coming decade from millions to billions around the globe.

**Fig. 1:**
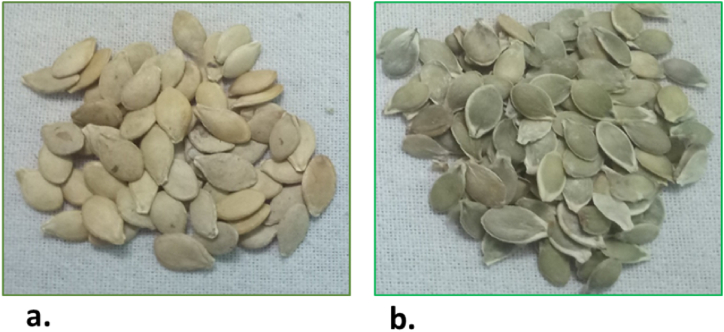
a. PCK-1 seeds b. PMK-1 seeds.

We hypothesize that the new cultivar of hull-less seeds of PMK-1 (Fig. 1b) genotype may function as an alternative supplement and bestow similar salubrious properties as that of hulled genotype PCK-1 (Fig. 1a), with the additional advantage of providing ease in oil extraction, sidelining the procedure of manual decortication resulting in attenuation of the farmer's cost. More importantly, a detailed biochemical characterization has not been performed on the new cultivar of hull-less seeds [13], therefore the present studies were conducted in lieu of comparative and comprehensive biochemical characterization of hulled and hull-less seeds. The studies can further be advanced to inter-disciplinary (biochemical, breeding, pharmacological) studies on improving the quality and attributes of the seeds to be used commercially in the agricultural, nutraceutical or pharmacological sectors.

## 2 Materials and methods

### 2.1 Collection of the sample

The pumpkin crops were grown at Vegetable Research Farm, Punjab Agricultural University, Ludhiana. The roots and shoots were harvested at fortnightly intervals from the day of transplanting, collected and stored at −80 °C for the enzymatic assay. The seeds were harvested at maturation of the crop, collected, dried, and stored at room temperature in the laboratory under airtight conditions.

### 2.2 Methods of analysis

#### 2.2.1 Determination of total soluble proteins

The quantitative estimation of total soluble proteins was determined by the method given by Ref. [22]. The ground seeds were extracted with 0.1 N NaOH and the sample-alkali mixture was centrifuged at 5000 rpm. The supernatant was collected, and the procedure was repeated thrice. The pooled extracts were subjected to 10% TCA precipitation, incubated for an hour, and centrifuged; the resulting pellet was dissolved in 0.1 N NaOH to form the final extract and the amount of total soluble proteins in the sample was measured using Bovine serum albumin as a standard.

#### 2.2.2 Determination of total free amino acids

The quantification of total free amino acids was determined using the method described by Ref. [23]. The extraction was initiated by refluxing seeds with 80% ethanol on water bath and the process was repeated thrice. Ninhydrin reagent was added to the extract aliquots, incubated for 20 min on the water bath, and then cooled at room temperature (25 °C). The absorbance was read at 570 nm using 80% ethanol as blank and quantitative estimation was done with the help of glycine as the standard.

#### 2.2.3 Determination of ash and moisture content

The ash and moisture content were analyzed by the procedure as recommended by the [24]. The samples of each genotype were incinerated at 600 °C for 6 h for ash content. For the moisture content, they were dried at 105 °C for 3 h and constant weight was achieved.

The calculations are as follows:

The loss in weight is calculated by (w2 – w1) – (w3 – w1).

Equation 1:

(1) $$\%\;Ash=\frac{W2-W3}{W1}X\;100$$

where

W1 = weight of sample.

W2 = weight of sample + crucible before drying.

W3 = weight of sample + crucible after drying to a constant weight.

The % moisture was calculated in similar way as that of % ash (Equation 1).

#### 2.2.4 Determination of fibre content

The fibre content was analyzed according to Ref. [25]. The seed powder was defatted with petroleum ether for 10 min and the defatted seed residue was subjected to acid hydrolysis followed by alkali hydrolysis. The neutralized residues were finally washed with 50% ethanol and transferred to pre-weighed silica crucibles (w1) and dried at 130 °C for 2 h. The crucibles containing residues were allowed to cool and then weighed (w2) and ashed in the muffle furnace for 4 h at 600 °C followed by cooling and weighing (w3). Calculations for % fibre follows the same calculation as that of % ash (Equation 1).

#### 2.2.5 Determination of DPPH radical scavenging activity

The antioxidant capacity was determined using method given by Ref. [26]. 2 mL of methanol was added to the ground seed powder and centrifuged at 2000 rpm for 10 min. The extract mixture containing 1 mL of sample and methanol along with 3 mL of 0.2 mM DPPH were mixed and incubated for 30 min in dark at room temperature. The DPPH radical scavenging activity was measured by reading the absorbance at 517 nm using methanol as control. The calculation is as given in Equation 2:

(2) $$DPPH\;scavenging\;activity\;\left(\%\right)=\frac{Absorbance\;of\;control\;–\;Absorbance\;of\;sample}{Absorbance\;of\;control}*100$$

#### 2.2.6 Determination of polyamines

Polyamines were extracted according to the [27] with modifications as mentioned ahead. In a volumetric flask, finely crushed seed powder was treated with 5% perchloric acid followed by incubation on ice for 30 min and then centrifuged at 27,000 rpm at 4 °C for 20 min. The resulting supernatant and pellet were hydrolyzed with 1 M NaOH. In 300 μL of alkali hydrolyzed samples, an equal volume of 12 M HCl was added and 110 °C for 16–18 h in the injectable vials. The carbonized material so obtained was separated and vacuumed and 300 μL of 5% perchloric acid was added. To procure the dansylamines, 200 μL samples were mixed with an equal proportion of 1, 7- diaminoheptane and incubated in dark at 60 °C for one hour proceeded by the addition of 100 μL of concentrated proline in dark for half an hour and extracted by vortexing using 500 μL toluene. After drying, 400 μL of dansylamines with 800 μL of acetonitrile were filtered through a 0.45 μm syringe filter and then finally injected into Agilent HPLC 1200 series Model no. 61311A. Reverse-phase column chromatography was carried out with the ODS Spheri-5 column at a flow rate of 5 mL/min. The mobile phase consisted of acetonitrile and water in the ratio of 70:30% at 0–4 min, 100:0% at 4–8 min and 70:30% at 8–20 min. The standards and samples were monitored using fluorescence detectors with excitation and emission at 252 and 500 nm, respectively.

#### 2.2.7 Determination of oil yield

The extraction of seed oil was carried out according to the method given by Ref. [24]. The oil extraction was done with the Soxhlet extraction unit. In the extractor device, 250 mL of petroleum ether and diethyl ether were used as solvents in a ratio of 2:3. The seed powder (in folded filter paper) was placed on the extractor device and the extraction was done for 8 h at solvent's boiling point (60 °C). Finally, the samples were dried in the oven at 100 °C for 3 h and were re-weighed to determine the content of oil.

#### 2.2.8 Determination of sterols

The content of sterols present in the seeds was accomplished by methodology given by Ref. [28]. The seed oil extract was diluted with chloroform and color-developing reagent (pre-cooled mixture of acetic anhydride and concentrated sulphuric acid) was added slowly. The test tubes were incubated in water bath at 16–18 °C for 10 min and rested in dark to cool as well as allow the color to develop. Finally, the absorbance was measured at 625 nm and the sterol content was assessed using cholesterol as a standard.

#### 2.2.9 Determination of fatty acid composition

The composition of fatty acids was profiled by the analytical method described by Ref. [29]. Fatty acids were transformed into fatty acid methyl esters (FAMEs) by mixing seed powder with petroleum ether and sodium ethoxide; water impurities were removed by addition of sodium chloride. The petroleum ether layer containing FAMEs was injected and analyzed in the Agilent technology GC model 7820A fitted with FID with the CP-Sil 88 25 m × 0.25 mm x 0.20 μm FAME column. The injection volume of FAMEs was 1 μL at a flame temperature between 230 and 240 °C, the Ionization detector temperature was 240 °C and oven temperature was between 180 and 200 °C. Nitrogen (N_2_) was used as a carrier gas at a flow rate of 60 mL/min, with the rate of flow of hydrogen and air at 30 mL/min each. The separation of volatile components occurred with the help of 50% cyanopropyl-methylpolysiloxane as a stationary phase of column and silica fused polyimide coating as a solid support of column. The concentration of the fatty acids was measured using EZ Chrome elite software.

#### 2.2.10 SDS-PAGE analysis

To profile proteins based on the molecular weight, the proteins were extracted from the defatted seed powder (obtained after the oil extraction according to Ref. [30] by sequential extraction according to the Osborne classification of storage proteins. The proteins were extracted by mixing the defatted seed powder in 0.7 mL deionized water followed by vortexing for 2 h and finally centrifuged at 13,500 rpm for 5 min at 4 °C. Four different extracts of seed proteins were obtained using this procedure of extraction with the aid of deionized water followed by 0.7 mL of 1 M NaCl, 0.5 M NaOH and 70% isopropanol, where the pellet was re-dissolved in the solvent for the extraction and the supernatant was kept for further use. All the extracts were stored at −70 °C prior to loading onto the gel. The separation of similar charged proteins by SDS-PAGE was done by the established protocol as referred by Ref. [31].

#### 2.2.11 Determination of tocopherols

The tocopherol content was determined using methodology provided by Ref. [32]. The extraction was initiated by homogenizing seeds in a pestle and mortar using absolute ethanol. The absorbance was read at 517 nm and the tocopherol content was measured using α-tocopherol as standard.

#### 2.2.12 Assay of specific activity of enzyme ACCase

The assay of specific activity of enzyme was accomplished by the extraction procedure according to Ref. [33] and the cues for the assay were taken from the methodology of [34]. Small pieces of roots and shoots were homogenized in pestle and mortar using 100 mM Tricine KOH buffer. The homogenate was filtered through muslin cloth and centrifuged at 15,000 rpm at 4 °C for 25 min. The resulting supernatant was kept for further use. To the reaction mixture, 100 mM potassium phosphate (pH 8) was added followed by the enzyme extract and the mixture was incubated at 30 °C for 5 min. The incubated aliquots were precipitated with 10% TCA on ice for 5 min and then centrifuged at 3000 rpm at 4 °C for 5 min. 50 μL supernatant was transferred into the cuvette containing 950 μL buffer (100 mM potassium phosphate (pH 8), 0.1 mg/mL DTNB, 20 mM oxaloacetate and 1 mg/mL BSA) and the absorbance was read at 412 nm. The concentration of remaining acetyl CoA was calculated using acetyl CoA as a standard.

### 2.3 Statistical analysis

Statistical analysis was performed for each biochemical parameter for samples in triplicate and data was presented as mean ± SD. The variance analysis and significant differences among the means were analyzed with one-way and two-way analysis of variance (ANOVA) by using CPCS software version 1.0. Significance was determined at p < 0.05.

## 3 Results and discussion

### 3.1 Total soluble proteins and total free amino acids

The content of total soluble proteins was quantified to be 3.48 ± 0.01 g per 100 g and 3.31 ± 0.01 g per 100 g seeds in hulled and hull-less genotypes, respectively (Table 1) [35]. reported protein content of 1.28% in *Cucurbita maxima* L. var. berrettina [36]. published 25.40% of protein content in *Cucurbita pepo* subspp. pepo var. styriaka. Although the protein content varies in context of species, location, crop practices and environmental factors, total protein content in range of 25.2–37% was observed by a plethora of authors [[37], [38], [39], [40], [41]].

**Table 1:** Biochemical profile of PCK-1 and PMK-1 genotype of pumpkin seeds.

Parameter	PCK-1	PMK-1	CV (5%)
Amino acids (mg/100 g)	79.62 ± 0.77	19.87 ± 0.35	1.20
Total soluble proteins (g/100 g)	3.48 ± 0.01	3.31 ± 0.01	0.27
Minerals (g/100 g)	3.66 ± 0.07	4.57 ± 0.2	3.66
Antioxidant capacity (%)	26.15 ± 0.47	20.95 ± 0.52	2.10
Polyamines (mg/100 g)	19.2	12.9	
Tocopherols (mg/100 g seed)	11.53 ± 0.16	15.76 ± 0.20	1.29
Tocopherols (mg/100 g seed oil)	0.38 ± 0.01	0.44 ± 0.01	2.88
Sterols (mg/100 g seed)	387.10 ± 2.58	260.65 ± 4.87	1.20
Sterols (mg/100 g seed oil)	12.64 ± 0.55	7.24 ± 0.02	3.94
Fibre (g/100 g)	21.1 ± 0.56	7.87 ± 0.15	2.82

The content of total free amino acids was observed to be 79.62 ± 0.77 mg/100 g and 19.87 ± 0.35 mg/100 g in hulled and hull-less seeds, respectively (Table 1). Soybean seeds (vegetable-cum-oilseed) were lauded up to few years for having protein quality (qualitative and quantitative) equal to that of animal and to possess eight out of overall nine essential amino acids [42,43], nevertheless pumpkin proteins are rich in all essential amino acids [18,44]. The results propound that pumpkin seeds are valuable protein source, and a thorough source post the oil extraction, adding to its advantage to be used commercially.

### 3.2 Ash, moisture and fibre content

Ash content analysis is a crucial part of the proximate analysis of food samples because it serves as a driving parameter in the determination of quality of the cattle and poultry feed material [45]. The hull-less and hulled genotype seeds were observed to possess 4.57 ± 0.2 g and 3.66 ± 0.07 g of total ash per 100 g dry seeds. The results are in line with [17,46,47]. The results are lower than those reported by Refs. [36,48] nevertheless are higher than reported by Refs. [35,44].

The moisture content of seeds relates to their shelf life and the percentage of moisture content is inversely proportional to the shelf life. The moisture content for both the hulled and hull-less seeds was quantified to be 6.74 ± 0.26% and 5.37 ± 0.15% respectively. The low moisture percentage of seeds of both genotypes indicates their high potential of long storage capacity and resistance from microbial or other parasitic attacks. The moisture (%) of seeds under study lies in the range observed by [46,49].

The fibre content of hulled seeds accounted to 21.1 ± 0.56 g per 100 g and was observed to be higher in comparison to the hull-less seeds i.e., 7.87 ± 0.15 g per 100 g (Table 1) and varies by 2.7-fold difference between the two. The fibre content in hulled seeded genotype goes well with the results published by Refs. [50,51] in the pumpkin fruit flour; higher than [48,52,53] reported % fibre in range of 11.69–24.85% and lower than reported by Ref. [54] in pumpkin rinds (14.83%) and pumpkin seeds (31.48%) in *C. pepo*.

### 3.3 DPPH free radicals scavenging activity

The antioxidant capacity in hulled and hull-less seeds was observed to be 26.15 ± 0.47% and 20.95 ± 0.52% respectively (Table 1). The antioxidant potential of the hull-less seeds accords with the results of [55]. [56] estimated antioxidant activity in ten genotypes of hull-less pumpkin seeds and found it varying in range of 0.193–11.753%.

### 3.4 Oil yield and sterols

The oil content was found to be 30.67 ± 1.22% in hulled seeds and 36 ± 0.6% in the hull-less seeds. The oil yield for the hulled seed oil parallels with the results published by Ref. [47], lies within the range as given by Ref. [49] for the three species of pumpkin seeds while [46] recorded a higher content of oil. The oil yield of both kinds of seeds lies in close proximity to the results given by Ref. [57]. [36,58] recorded higher content of oil in the naked seeds.

The sterol content found in the hulled and hull-less genotypes accounted to 387.10 ± 2.58 mg/100 g DW and 260.65 ± 4.87 mg/100 g DW seeds respectively; 12.64 ± 0.55 mg/100 g seed oil in hulled and 7.24 ± 0.02 mg/100 g in hull-less seed oil, respectively (Table 1). Observations made in the present study accorded with the results published by [38.59.60] and in near agreement with [53].

### 3.5 Tocopherols

The total tocopherol content in the hull-less or naked seeded genotype (PMK-1) was quantified as 15.76 ± 0.20 mg/100 g seed, 0.44 ± 0.01 mg/100 g seed oil and 11.53 ± 0.16 mg/100 g seed, 0.38 ± 0.01 mg/100 g seed oil in the hulled seeded genotype (PCK-1) (Table 1). The results equate with the results published by Ref. [53]. [60] obtained much higher results in the husked and naked seed oil. As per the tocopherol content in the husked and naked pumpkin seed oil [59,61] reported contrasting results [59]. reported 52mg/100 g in the hull-less seeds and 70.9 mg/100 g in the husked seeds [61]. reported 42.27 mg/100 g and 67.56 mg/100 g in naked and husked seeds respectively.

### 3.6 Composition of polyamines

As given in Table 2, the content of spermidine was present in highest content followed by putrescine followed by spermine in both kinds of seeds. The total content of three polyamines (PAs) in hulled seeded genotype is found to be more than hull-less seeded genotype. The difference can be due to the genotypic variations and the presence of thick seed coat in one genotype. The results are found to be higher than reported for putrescine and lie within the values for spermine as given by Ref. [62]. A substantial high amount of the three PAs were reported by Ref. [63].

**Table 2:** Quantitative values of polyamines.

Polyamine (mg/100 g)	Hulled seeds (PCK-1)	Hull-less seeds (PMK-1)
Spermidine	10.4	7.9
Putrescine	5.6	3.2
Spermine	3.2	1.8
**Sum**	19.2	12.9

**Fig. 2:**
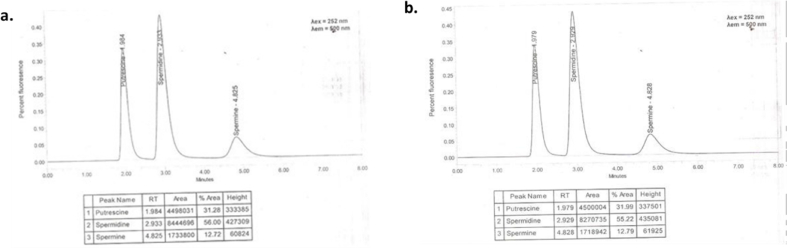
a.HPLC Chromatogram of polyamines in PCK-1 seeds b. HPLC Chromatogram of polyamines in PMK-1 seeds.

The HPLC chromatograms (Fig. 2a and Fig. 2b) did not only detail the quantity of the PAs present, also facilitated in detailing the absence of any other PAs in the pumpkin seeds under study. The significant presence of the PAs as determined by the HPLC chromatography suggested the role of seeds as potential source of strong antioxidants, where amongst the PAs, spermine is believed to be the most effective antioxidant. PAs and their metabolites also function as biological markers for cancer. In plants, PAs play a diversity of roles in various physiological pathways like embryogenesis, flower development, fruit development, senescence and in various abiotic and biotic stresses [[64], [65], [66]].

### 3.7 Composition of fatty acids

Pumpkin seed oil was found to be comprised of four dominant fatty acids viz. palmitic acid (16:0), stearic acid (18:0), oleic acid (18:1) and linoleic acid (18:2). The saturated fatty acids palmitic and stearic acids were found to be 8.58% and 2.86% in the hulled genotype; 9.5% and 3.74% in the hull-less genotype seed oil. In the hulled seed oil, the unsaturated fatty acids, oleic acid, and linoleic acid quantified to 46.27% and 42.29%, and forms 88.56% of total unsaturation. Whereas in the hull-less genotype seed oil, palmitic and stearic acids were found to be 9.5% and 3.74%; 46.88% and 39.88% of oleic acid and linoleic acid respectively, making 86.76% of total unsaturation (Table 3). The fatty acid profile is in accordance with published fatty acid profile by plethora of authors i.e., [17,36,57,59,61,[67], [68], [69]].

**Table 3:** Composition of fatty acids in PCK-1 and PMK-1 genotypes of pumpkin seeds.

Fatty acid (%)	PCK-1	PMK-1
Palmitic acid (16:0)	8.58^a^	9.5^a^
Stearic acid (18:0)	2.86^b^	3.74^b^
Oleic acid (18:1)	46.27^c^	46.88^c^
Linoleic acid (18:2)	42.29^d^	39.88^d^
Saturated fatty acids (%)	11.44	13.24
Unsaturated fatty acids (%)	88.56	86.76

a,b,c,d- There is no significant difference in the fatty acid composition amongst the two genotypes.

**Fig. 3:**
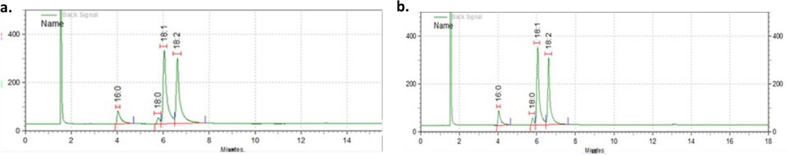
a.GLC Chromatogram of PCK-1 seeds b. GLC Chromatogram of PMK-1 seeds.

The GLC chromatogram (Fig. 3a and b) clearly indicates the presence of only four fatty acids in the seed oil. The high percentage of unsaturation of the pumpkin seed oil contributes to the stabilization of the oil as well as making it nutritionally desirable.

### 3.8 Profiling of proteins by SDS-PAGE

**Fig. 4:**
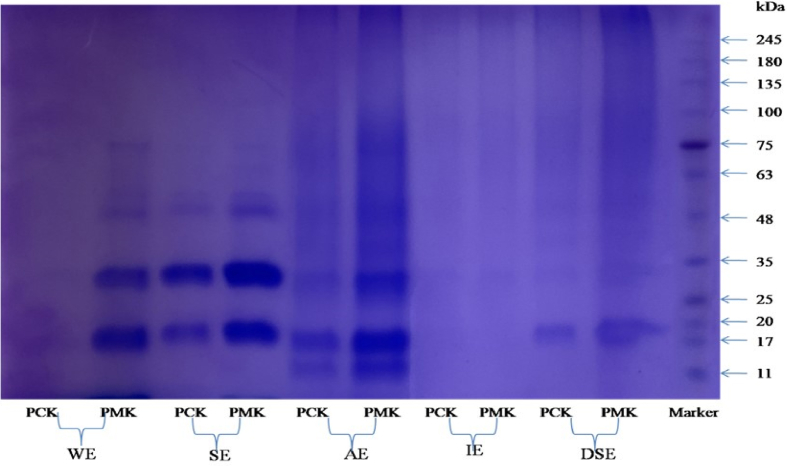
Eletrophoretic pattern of proteins of defatted pumpkin seed flour under reducing conditions. WE-water extract; SE-salt extract; AE-alkali extract; IE-Isopropanol extract; DSE-Defatted seed extract.

As the proteins form pertinent bioactive molecules, therefore, an electrophoretic protein profile facilitates obtaining the possibilities of disparate types of proteins, range of molecular weight, presence of any distinct kind of protein. The SDS-PAGE profile can be observed in Fig. 4, and it was seen that the protein size in all the sample extracts varied from 11 to 75 kDa. In water extracts of PCK-1 and PMK-1, the protein bands varied from 17 to 75 kDa protein sub-units. In salt extracts, the size of globulins ranged from 20 to 48 kDa. The protein bands were similar among the two genotypes viz. there is no disparity in the globulin proteins in the hulled and hull-less seeds. The protein bands of albumin and globulin fractions can also be observed as similar to each other. In the alkali extracts of proteins, an additional protein is discerned in parallel to 11 kDa sub-unit of marker protein, and no considerable distinction was visible in both genotypes. In the alcoholic extracts, no intense bands were visibly observed but on scrutiny, a single band was slightly visible amid 25–35 kDa sub-units. In the residual defatted seed extracts, only one protein band was discerned, where the size of the band is equivalent to 17–20 kDa subunits, indicating that there might be no protein left in the residual defatted protein extract after the sequential extraction. The two genotypes do not differ considerably with respect to the composition of seed storage proteins.

The SDS-PAGE evaluation of the proteins of hulled and hull-less seeds accords with the observations made by Ref. [70]. According to the reports of [71], the 12 kDa protein fraction is difficult to precipitate during the fractionation of proteins, however [70] reported a single intense band of molecular weight (13–14 kDa) in the glutelins (alkali extract). The results obtained from the electrophoretic pattern of proteins under reducing conditions align with those of [72,73].

### 3.9 Specific activity of acetyl CoA carboxylase (ACCase)

The enzyme Acetyl CoA Carboxylase (EC 6.4.1.2) catalyzes the first committed step of the conversion of acetyl CoA to malonyl CoA during biosynthesis of fatty acids [33].

The values given in Table 4 delineate the specific activity of ACCase in the range from 309.36 to 635.34 units in the hulled genotype and varies from 290.80 to 594.83 units in the hull-less genotype. The specific activity decreased progressively from the first stage to the last. The average specific activity of ACCase in the roots of hulled genotype (PCK-1) accounts as 421.36 mM of malonyl CoA formed/min/mg protein and 402.08 mM of malonyl CoA formed/min/mg protein in the roots of hull-less genotype (PMK-1).

**Table 4:** Specific activity (mM of malonyl CoA formed/min/mg protein) of enzyme in roots of PCK-1 and PMK-1 genotype.

Stage	Hulled genotype (PCK-1)	Hull-less genotype (PMK-1)
I	635.34 ± 28.65	594.83 ± 4.66
II	452.83 ± 7.55	442.15 ± 6.88
III	383.75 ± 8.47	371.78 ± 3.68
IV	325.53 ± 10.39	310.83 ± 1.28
V	309.36 ± 13.13	290.80 ± 1.94
Mean	421.36	402.08
CD (5%)	A (Stages of development) = 3.77 B (Genotypes) = 5.95 A*B = 8.42	

I, II, III, IV and V represent the stages of development from the time of harvesting and the experiment calculations were performed in triplicate. The calculations were statistically calculated using 2-way ANOVA and values are represented as mean ± standard deviation.

As elucidated in Table 5, the specific activity of ACCase in the shoots of PCK-1 can be observed to be in range of 346.28–807.94 units and 315.33–696.32 units in PMK-1 shoots. The enzymatic pattern is similar to the one found in roots. The average specific activity of ACCase in the roots of PCK-1 is 493.68 mM of malonyl CoA formed/min/mg protein and 445.38 mM of malonyl CoA formed/min/mg protein in case of PMK-1.

**Table 5:** Specific activity (mM of malonyl CoA formed/min/mg protein) of enzyme in shoots of PCK-1 and PMK-1 genotypes.

Stage	Hulled genotype (PCK-1)	Hull-less genotype (PMK-1)
I	807.94 ± 14.96	696.32 ± 17.11
II	534.13 ± 3.79	479.37 ± 5.27
III	423.51 ± 4.11	394.20 ± 5.46
IV	356.56 ± 1.68	341.69 ± 4.10
V	346.28 ± 7.35	315.33 ± 2.64
Mean	493.68	445.38
CD (5%)	A (Stages of development) = 6.31 B (Genotypes) = 9.98 A*B = 14.11	

I, II, III, IV and V represent the stages of development from the time of harvesting and the experiment calculations were performed in triplicate. The calculations were statistically calculated using 2-way ANOVA and values are represented as mean ± standard deviation.

The specific activity of the enzyme ACCase provides value to the pumpkin plants (often discarded as waste) as an unconventional raw material for study of the target enzyme and designing of the enzyme inhibition drugs as a potential source in the treatment of chronic diseases like obesity, diabetes, and cancer.

Overall, we observed that the biochemical composition of the two genotypes were nearly similar to each other barring the higher fold difference found for total free amino acids and the fibre content in the hulled seeds relative to hull-less seeds. Interestingly, these differences can be attributed largely to the presence and the composition of the hulls, along with the genotypic variation.

## 4 Conclusion

The conducted study has demonstrated pumpkin seeds as an unconventional source of functional food, being a prolific source of numerous imperative nutrients (essential for the healthy state of a living being) like proteins, lipids, minerals, antioxidants, fatty acids, fibre, sterols, and polyamines. The hulled genotype of pumpkin seeds viz. PCK-1 was observed to possess higher content of total soluble proteins, amino acids, antioxidant capacity, polyamines, sterols, and fibre content whereas the hull-less genotype viz. PMK-1 was observed to possess higher amount of minerals, tocopherols, and oil content. There was no significant difference in the protein profile amongst the two genotypes. The specific activity of enzyme was significantly different with respect to genotype as well as the developmental stage. The rich nutritious profile of seeds is a boon for the substantial proportion of the population as it serves as a substitute for the expensive protein supplements especially for the young and old, pregnant, or lactating females who require more protein for the balanced functioning of the body.

The crucial roles of metabolites like polyamines which function as markers of cancer in the physiological system, higher content of unsaturated fatty acids viz. oleic and linoleic acids confers higher stability to the oil as well as prolific source of ω-fatty acids, tocopherols acting as antioxidants models the pumpkin seeds as a whole nutritious food and must be encouraged to consume by a larger population across the globe oblivious to the nutritive properties of the seeds.

Besides, the unique trait of the seeds being hull-less, is vital from the commercial point of view as it plays a key role in reducing farmers’ expenditure on decortication process. The biochemical make-up of these vegetable-cum-oilseeds renders them the importance from distinct perspectives of research like breeding, nutrition, food technology, therapeutics, pharmaceutics, cosmetics, and other industrial applications for their further commercial exploitation.

The limitations of the present study adhere that the biochemical composition, may vary with respect to the soil conditions, weather as well as the cultivating conditions along with other agricultural practices of the crop, which may bring further changes to the composition values in the cultivars. Additionally, we cannot ignore the fact that the advantages of hull-less cultivar comes at its own expense that it leads to the loss of substantial amount of fibre, which can be compensated in the dietary supplements for humans but is irreplaceable for other purports.

## Author contribution statement

Ananaya Charaya: Conceived and designed the experiments; Performed the experiments; Wrote the paper.

Neena Chawla: Conceived and designed the experiments; Contributed reagents, materials, analysis tools or data; Wrote the paper.

Ajmer Singh Dhatt, Madhu Sharma: Contributed reagents, materials, analysis tools or data.

Sanjula Sharma: Performed the experiments, data analysis.

Inderpal Kaur: Analyzed and interpreted the data.

## Funding statement

This research did not receive any specific grant from funding agencies in the public, commercial, or not-for-profit sectors.

## Data availability statement

Data included in article/supplementary material/referenced in article.

## Declaration of interest’s statement

The authors declare no competing interests.
